# How to conjugate the stemness marker ALDH1A1 with tumor angiogenesis, progression, and drug resistance

**DOI:** 10.20517/cdr.2019.70

**Published:** 2020-03-19

**Authors:** Valerio Ciccone, Lucia Morbidelli, Marina Ziche, Sandra Donnini

**Affiliations:** ^1^Department of Life Sciences, University of Siena, Siena 53100, Italy.; ^2^Department of Medicine, Surgery and Neuroscience, University of Siena, Siena 53100, Italy.

**Keywords:** Aldehyde dehydrogenase 1A1, angiogenesis, drug resistance, immune editing, metastases, stemness

## Abstract

Cancer is the second leading cause of death worldwide. The survival of cancer patients depends on the efficacy of therapies and the development of resistance. There are many mechanisms involved in the acquisition of drug resistance by cancer cells, including the acquisition of stem-like features. Cancer stem cells (CSCs) represent a major source of tumor progression and treatment resistance. CSCs are a subpopulation of cancer cells having the abilities to self-renew and form spheres *in vitro*. Aldehyde dehydrogenase 1A1 (ALDH1A1) is a cytosolic enzyme involved in the detoxification of cells from toxic aldehydes and belongs to the ALDH family. High ALDH1A1 activity is closely related to stemness phenotype of several tumors, possibly contributing to cancer progression and diffusion in the body. We have documented the contribution of ALDH1A1 in tumor angiogenesis in breast cancer cells by the activation of hypoxia inducible factor-1α and vascular endothelial growth factor signaling. This review discusses the involvement of ALDH1A1 in the development of different hallmarks of cancer to propose it as a novel putative target for cancer treatment to achieve better outcome. Here, we analyze the involvement of ALDH1A1 in the acquisition of stemness phenotype in tumor cells, the regulation of tumor angiogenesis and metastases, and the acquisition of anticancer drug resistance and immune evasion.

## Introduction

Cancer is highly dynamic as tumor cells become more heterogeneous during the progression of the disease. Twenty years ago, Hanahan and Weinberg published a milestone review, in which they organized the complexity of tumor biology into six major hallmarks, including cell stemness^[1]^. An updated review from 2011 by the same authors added two emerging hallmarks: reprogramming of energy metabolism and evading immune destruction. The translation of hallmarks into clinical practice has been proved by the development of drugs associated with specific hallmarks^[2]^, but the view of their complementarity is necessary to explain the heterogeneity of cancer^[3]^.

Tumor cells in the neoplastic mass, characterized by enhanced capacity of self-renewal and elevated ability to seed new tumors upon injection into new hosts, are termed cancer stem cells (CSCs). For these features, CSCs have also been referred to as “tumor-initiating cells” or “sphere-forming cells”. CSCs can be implicated in many hallmarks of cancer and recently CSC-directed therapies have been clinically developed^[4]^. The involvement of these cells in chemoresistance is known, in particular by the maintenance in G0 phase; high expression of drug efflux pump, anti-apoptotic proteins, and scavenger molecules; more efficient DNA repair; and a protective microenvironment created by interaction with the tumor niche^[5,6]^.

The gold standard model to define CSCs is the serial dilution *in vivo* transplantation^[7]^, but several markers of cell surface and intracellular compartment have been identified. Aldehyde dehydrogenase 1A1 (ALDH1A1) is a protein that has been used to mark CSCs in several cancers, including leukemias and carcinomas of the breast, colon, liver, lung and pancreas, among others. This enzyme belongs to the ALDH superfamily, composed of nicotinamide adenine dinucleotide (phosphate) (NAD(P)+)-dependent enzymes that catalyze the oxidation of exogenous and endogenous aldehydes to the respective carboxylic acids.

Overproduction of reactive oxygen species (ROS) in cells may cause oxidation of polyunsaturated fatty acids in the cellular membrane through free radical chain reactions with the formation of aldehydes as final products, which play a crucial role in the pathogenesis of various chronic and acute diseases such as cancer, inflammation, and atherosclerosis, among others^[8,9]^. However, aldehydes are ubiquitously distributed in the environment and hence they can be acquired from different sources such as food, water, and air^[10]^. By preventing the accumulation of aldehydes derived from endogenous or exogenous processes, ALDHs mitigate oxidative damage at the cellular and tissue levels. In humans, there are 19 isoforms of ALDH with different cell compartmentalizations and specific tissue distributions. The ALDH1 family, especially ALDH1A1 and ALDH1A3 isoforms, is required for retinoic acid (RA) biosynthesis. The role of ALDH1A1 as robust marker of stemness has been reported in depth by several authors, but in the last decade the emerging role in tumor progression of this enzyme has been proposed^[11]^. Tumor cells are characterized by an aberrant redox state and an increase of ALDH1A1 expression can be helpful as ROS scavenger. Further, the metabolic activity of ALDH1A1 is strictly related to inactivation of anti-cancer drugs, including oxazaphosphorines or aldophosphamide, in nontoxic metabolite and the expression of this enzyme is a marker of poor prognosis in many tumors^[12]^. The silencing of the ALDH1A1 gene in human breast cancer cells increases their sensitivity to paclitaxel with a concomitant increase of ROS formation, and similar results were obtained with doxorubicin, sorafenib, and staurosporine^[13]^. The opposite effect is observed when ALDH1A1 is present. It has been proposed that cancer cells acquire drug resistance by inducing ALDH1A1 expression^[14]^. Indeed, increased ALDH1A1 expression in relapsing tumors of human breast cancer after surgery and tamoxifen treatment has been observed^[15]^. ALDH1A1 expression in breast cancer has also been associated with advanced disease stages, triple negative cells, and poor prognosis^[16]^. Although several biological functions are mediated by RA, the molecular mechanism by which ALDH1A1 regulates cellular functions in oncology is not clear.

In the present review, we describe the contribution of ALDH1A1 in tumor stemness and cancer progression, analyzing its involvement in angiogenesis, tumor spread, drug-resistance, and immune-evasion.

### Methodology

A search was conducted using the terms listed in Table 1 in the PubMed bibliographic database, including original articles, reviews, and clinical data belonging to the experimental area, written in English, and published from January 2000 to September 2019 in journals with high impact factors. Useful papers published before January 2000 were also included. We performed a narrative synthesis of the results reported in the selected manuscripts.

**Table 1:** Main and secondary keywords used for literary search

Main key words	Secondary key words
ALDH1A1 Cancer Tumor Neoplasia Tumor microenvironment	Preclinical study Clinical study Stemness Drug resistance Proliferation Migration Invasion Vitality Survival Apoptosis Angiogenesis Metastases Immuno evasion

Secondary key words were utilized in combination (by using “AND”) with the main key words. The main keywords were combined by using “OR” and are reported in left column. ALDH1A1: Aldehyde dehydrogenase 1A1

### CSCs

Cancer is the result of oncogenic mutations with survival advantages over other cells^[17]^. During tumorigenesis, any cell is susceptible to malignant transformation depending on the accumulation of oncogenic mutations. Recently, it has been recognized that the majority of cancers are due to random mutations arising during DNA replication in stem cells (SCs) and only a third are attributable to environmental factors or inherited predispositions^[18]^.

In the last years, the concept of CSCs has received increasing interest in tumor progression and regeneration. It is thought that cancer recurrence could be mediated by these CSCs. They are a subpopulation of cancer cells having the ability of self-renewal as well as tumor-initiating capacity. In addition, they are now recognized to play a crucial role in tumor metastases, relapse, and chemo/radio-resistance^[19,20]^. Indeed, cancer cells with stem phenotype acquire a quiescent state, cell cycle arrest, and lower response to chemotherapeutic agents^[21,22]^, as well as to novel therapies such as immunotherapy and anti-angiogenics^[23]^. As tumor initiators, CSCs are considered to be a promising target for obtaining a better therapeutic outcome. Nowadays, cancer treatment has been reoriented toward the understanding of CSCs’ intrinsic properties and mechanisms that they develop to survive and enhance their aggressive phenotype.

The discovery of CSC markers has facilitated their isolation and characterization. They have been identified in blood, breast, colon, melanoma, pancreatic, prostate, ovarian, and lung cancers, among others. In recent years, many molecules have been proposed as CSC biomarkers. More than 25 transcriptional factors codify for surface and intracellular stemness markers. CD44 is a transmembrane glycoprotein that plays an important role in cell division, migration, adhesion, and signaling. It is expressed in many tumors, including bladder, breast, colon, gastric, glioma, head and neck, osteosarcoma, ovarian, pancreatic, and prostate cancers, as well as leukemia^[24]^. CD133 is another transmembrane glycoprotein localized in cellular protrusions that regulates stem-like features of many cancers. In addition, several stemness markers are not localized on the cell surface. ALDH1A1 is a robust marker of these cells in many tumors and it could be used to enrich tumor-initiating subpopulations from various cell lines and primary tumors^[25-30]^.

### ALDH1A1

ALDH1A1 belongs to the ALDH1 family and is a marker of normal SCs and CSCs^[31]^. In cell cultures, the activity of this class of enzymes is measured by Aldefluor kit that determines the total activity of ALDH1 family. Fluorescent substrate of enzymes are used to quantify ALDH activity. This probe is retained in the cells and the amount of fluorescent reaction product, measured with flow cytometer, is proportional to ALDH activity. ALDH1A1 activity is associated to poor clinical outcome in lung, esophagus, stomach, ovarian, breast and colorectal cancer patients^[26,32-36]^. ALDH1A1 is a cytosolic enzyme involved in detoxification of acetaldehyde deriving from ethanol metabolism, similar to ALDH2, which is preferentially located in mitochondria. These enzymes are mainly responsible for acetate production in the cells and counteract alcohol-related carcinogenesis^[37]^. Moreover, the ALDH1 family catalyzes the formation of RA from retinaldehyde. Retinoic acid binds retinoic acid receptor or retinoic X receptor, which transcriptionally regulates many downstream developmental genes in physiological and pathological conditions^[38,39]^. Indeed, retinoids are involved in the pathogenesis of cancer, obesity, diabetes, and cardiovascular diseases, where the metabolism of these compounds is strictly regulated by the activity of the ALDH1 family, specifically isoforms A1-A3. Luo *et al*.^[40]^ demonstrated that the RA-target genes and RA responsive elements are characteristic traits of melanoma CSCs from patients. ALDH1A1 can also maintain CSC phenotype by preventing apoptosis through ROS level regulation^[41]^.

Besides the catalytic activity, ALDH1A1 exerts many biological functions via non-catalytic pathways, for example through the binding to hormones (e.g., androgen, thyroid hormone, and cholesterol), small molecules, and xenobiotics (e.g., daunorubicin and quinolone)^[42]^. However, ALDH1 is mostly involved in stemness phenotype of tumor through its catalytic activity. CSCs exhibit elevated ALDH1A1 catalytic activity, and metabolic inactivation of selected anti-cancer agents by such activity is believed to underlie chemoresistance and consequent cancer recurrence.

## ALDH and cancer

In this section, we describe the effects of ALDH1A1 in stemness phenotype tumor angiogenesis, tumor metastases, antitumor drug resistance, and immune evasion, hypothesizing its central role in tumor malignancy.

### ALDH and stemness

The transformation of SCs into CSCs, cancer progenitor cells, is an important step in carcinogenesis as, after CSC development, they remodel the tissue niche and produce a pathological cancer microenvironment termed “CSC niche”. The CSC niche is responsible of four main functions, namely anchorage, survival, protection, and proliferation, in addition to the maintenance of cancer stemness and plasticity and the editing of the immune system^[43]^. Cells composing the CSC niche are important for CSC maintenance and the generation of factors further involved in invasion, metastases, and promotion of angiogenesis^[44-46]^. ALDH1A1 is historically considered a pivotal regulator of CSCs. In 2014, Menendez and Alarcon coined the term “metabostemness” to indicate the orchestration of the genetic reprogramming that redirects normal and tumor cells toward less-differentiated CSC states^[47]^. The stemness state is not permanent because there is a dynamic and bidirectional interconversion between CSCs and non-CSCs, depending on their relative abundance^[2]^; in other words, not only can cancer cells become CSCs, but the reverse process can also happen. Accordingly, ALDH1A1 is a CSC marker of many tumors, but it is also expressed in tumor cells in a more differentiated state, characterized by lower or limited proliferative potential^[48,49]^. This plasticity may depend on the niche where CSCs live. The CSC niche is represented with a specific microenvironment that could decide the fate of CSCs and be an important way to overcome cellular resistance and prevent metastases^[50]^. Despite accumulating evidence on the functional role of ALDH1A1 in stemness, the specific mechanisms involved in its regulation in CSCs remain unclear^[51]^. In recent years, different *in vitro* assays have been developed to evaluate stemness phenotype and metastatic potential of tumor cells^[52-54]^. In particular, transwell assay and tumorsphere growth in ultra-low attachment plates are correlated to tumorigenicity and stemness. Identification of CSCs by measuring ALDH1A1 activity has been analyzed by Aldefluor assay^®^. However, this test also identifies ALDH1A2 and ALDH1A3 isoforms^[31,39]^, suggesting a contribution of these other isoforms in the stem-like features of tumor cells. Since the ALDH superfamily comprises many isozymes characterized by specific tissue distribution, it is possible to speculate that different ALDH isozymes contribute to the maintenance of CSCs in different cancers. This speculation is supported by several pieces of evidence, demonstrating a specific elevated expression of ALDH1B1 in colon cancer^[55]^; ALDH3B1 in lung, breast, ovarian, and colon cancer^[56]^; ALDH3A1 in lung cancer^[57]^; and ALDH7A1 in prostate cancer^[58]^. Such information is critical for understanding the biological significance of the ALDH isozymes in the tumor cells and, as a consequence, for the development of anti-cancer therapies targeting ALDHs.

ALDH1A1 has recently been positively correlated with several *in vitro* tumor cell behaviors that are surrogates of cancer progression. Yang *et al*.^[59]^ demonstrated that acquisition of invasive and metastatic capabilities correlated with epithelial-mesenchymal transition phenotype in ALDH1A1^*high*^ esophageal squamous cell carcinoma. Consistently, ALDH1A1^*high*^ glioma cells showed higher rate of proliferation and colony formation compared to the same ALDH1A1^*low*^ cells^[60]^. Finally, overlapping results have been obtained in human breast cancer cells, in which the expression of ALDH1A1 was involved in the regulation of adhesion, migration, and colony formation and in the acquisition of metastatic phenotype^[61]^. Likewise, experimental studies have demonstrated that CSCs with high ALDH activity identified by Aldefluor assay^®^ showed high tumorigenic phenotype *in vitro* and *in vivo*. Moreover, the prognostic value of ALDH1A1 in cancers has been validated by using immunohistochemistry (IHC) on cancer patient tissues. ALDH1A1 overexpression evaluated by IHC has been correlated with poor prognosis in many cancers such as lung, ovarian, stomach, breast, and colorectal cancer. Various authors reported the prognostic value of ALDH1A1 in breast cancer patients. It seems to be associated with larger tumor size, lymph node infiltration, and poor outcome in systematic review and meta-analysis^[16,62]^. However, controversial data have been described in other tumors such as pancreas adenocarcinoma^[63,64]^ and malignant melanoma^[65,66]^. Explaining these discrepancies is not easy, but type of cancer and degree of maturation could be reasonable causes.

### ALDH and tumor angiogenesis

The remodeling of the tumor microenvironment (TME) in which CSCs reside is related to their ability to drive tumor growth and metastases^[67]^. TME is an integral part of the physiology, structure, and functions of CSCs, conditioning the fate of tumor cells in each state of differentiation. On the one hand, by providing various paracrine factors, TME plays a crucial role in maintaining CSC plasticity, by regulating pathways or transcription factors involved in self-renewal process. On the other hand, CSCs can actively recruit some of these niche components to create a microenvironment that is favorable for its survival. For example, CSCs can secrete vascular endothelial growth factor (VEGF) or other factors to recruit perivascular cells. TME consists in multiple types of cells such as endothelial cells, fibroblasts, mast cells, neutrophils, perivascular cells, adipocytes, macrophages, and immune-suppressive cells^[68]^. The contribution of stromal cells in the regulation of tumor hallmarks is now recognized, and the most important role of endothelial cells on tumor growth and the expansion of self-renewing CSCs, through the neovascularisation, is well-established^[69]^. An important observation by Balic *et al*.^[70]^ is the greater proportion of CSCs present in metastatic tumors compared to the primary site. The process known as “angiogenic switch” reflects increased bioavailability of growth factors for vascular cells, allowing the removal of cellular waste products^[71]^. The regulation of angiogenic phenotype by CSCs in many tumors is widely reported in the literature. Bao *et al*.^[72]^ demonstrated that stem-like glioma cells (CD133+) from human glioblastoma biopsy developed tumors having extensive angiogenesis in a VEGF-dependent manner. ALDH1A1 activates retinoic acid signaling, which, in turn, regulates hypoxia inducible factor-1α and the VEGF axis^[73,74]^. Lv *et al*.^[75]^ obtained similar results in patients with invasive ductal breast carcinoma expressing ALDH1 enzyme. Many researchers obtained overlapping results in other tumor types. Silva *et al*.^[76]^ suggested a potential control of angiogenic ovarian cancer based on ALDH activity and CD133 expression. It has also been reported that tumor endothelial cells showed increased expression of VEGFR2 and a highly angiogenic phenotype compared to normal endothelial cells. This behavior was related to ALDH1A1 expression in the same endothelial cells^[77]^. Finally, in a co-culture system of human stem cells from bone marrow, ALDH1A1^*high*^ cells promote endothelial cell organization on Matrigel^[78]^.

### ALDH and tumor metastases

Angiogenesis is required for tumor growth and metastases since new aberrant vessels are easily permeable to highly invasive tumor cells. Metastasis represents the main problem for cancer therapy. The possibility that cancer cells can switch into CSCs under specific conditions dictated by a depletion in the number of CSCs or by environment can open a new perspective on the metastatic process^[50]^. There is growing evidence supporting the role of ALDH1A1 in cancer invasive phenotype and the ability to metastasize. Its expression has been reported to correlate with increased *in vitro* migration in NSCLC^[79]^, renal^[80]^, esophageal^[79]^, and breast cancer cells^[61]^. Summarizing, increasing evidence from *in vitro* studies offers a mechanistic role for ALDH1A1 in metastases. Likewise, functional association of ALDH1A1 and *in vivo* metastases has been suggested. Ginestier’s group found high ALDH activity in cell lines derived from mammary tissue^[26]^. The same cells with high ALDH activity (Aldefluor-positive) showed stemness phenotype and metastatic potential. This was the first observation of ALDH activity in tumor stemness and progression^[26]^. Subsequent studies have shown that ALDH1A1 was involved in increased metastatic capability of prostate^[81,82]^, breast^[83]^, colon^[84]^, ovarian^[85]^, and esophageal cancer^[86]^, suggesting that the activity of the enzyme also has a functional role in more differentiated cancer cells, as well as in CSCs^[48,49]^. It has also been reported that depletion of ALDH1A1 by shRNA in melanoma cells resulted in significant delay in appearance of xenografted melanoma and reduction in tumor growth, as well as a decrease of metastases number after tail vein injection in mice^[87]^.

### ALDH and antitumor drug resistance

Besides cancer progression and invasion, CSCs are suggested to be responsible for drug resistance, and cancer recurrence can be due in part to their ability to renew themselves and differentiate into a heterogeneous lineage of cancer cells. The mechanisms by which CSCs escape radio- and chemotherapy include epithelial-to-mesenchymal transition, multidrug resistance, dormancy, and tumor microenvironment composition. Enhanced ALDH1A1 is a putative marker of resistance to chemotherapeutic agents, as well as immunotherapy and antiangiogenic strategies. This is not surprising because of the cellular protective role of this enzyme against various harmful aldehydes. In 1984, Hilton observed a chemoresistance role for ALDH in leukemic stem cells against cyclophosphamide, an alkylating agent^[88]^. ALDH metabolizes cyclophosphamide to 4-hydroperoxycyclophosphamide, an inactive excretory product. He found a reversion of cyclophosphamide resistance by inhibiting ALDH activity with disulfiram. Subsequent studies showed that ALDH1 could confer resistance in other cancers such as breast cancer^[89]^. The role of ALDH1A1 in drug metabolism has been demonstrated in a pharmacogenetic analysis. In 882 breast cancer patients treated with cyclophosphamide and doxorubicin, two SNPs of ALDH1A1 associated with an increased risk of hematological toxicity, grades 3 and 4, have been discovered^[90]^. In addition to conferring resistance to cyclophosphamide, ALDH1A1 knockdown experiments demonstrated its role in pancreatic adenocarcinoma cells sensitized to the gemcitabine effect^[91]^. Kulsum *et al*.^[92]^ showed that, in head and neck cancer cell lines resistant to cisplatin and 5-fluorouracil, there was an enrichment of ALDH1A1 overexpressing cells as compared to parental cells.

Recently, an ALDH1A1-induced resistance has also been found against tyrosine kinase inhibitors in *in vitro* and *in vivo* models of lung adenocarcinoma through the upregulation of superoxide dismutase and glutathione peroxidase 4 as well as ALDH1A1 itself, key enzymes involved in the redox signaling^[93]^. An upregulation of ALDH1A1 expression in non-small cell lung cancer resistant to cisplatin can be a consequence of sex-determining region Y-box-9 activation^[94]^. On the contrary, ALDH1A1 depletion could reverse cisplatin resistance in human lung cancer cells^[95]^.

In ovarian cancer, Kaipio *et al*.^[96]^ observed that ALDH1A1 was increased after taxane and platinum treatment and its expression is correlated with chemoresistance and reduced survival. In the same tumors cells, Świerczewska *et al*.^[97]^ showed that high levels of ALDH1A1 are linked to resistance to cytotoxic agents through protein tyrosine phosphatase receptor type K (PTPRK) downregulation.

Furthermore, Mori *et al*.^[98]^ demonstrated in a model of spheroid cells derived from human endometrial cancer that ALDH1A1 inhibition synergized with paclitaxel to block cancer proliferation.

The role of ALDH1A1 in mediating radioresistance in prostate cancer cells was proposed by Cojoc *et al*.^[99]^. They demonstrated that inhibition of Wnt pathway leads to a reduction of ALDH positive population and restores the response to radiotherapy.

Increased expression of stem cell markers correlated with enhanced stem-like features, migration, and *in vivo* tumor burden, demonstrating that chemoresistance, stemness, and metastases are strictly interwoven^[92]^. The acquisition of a quiescent state from CSCs could be due to the selection pressure imposed by chemotherapy. It is primarily due to slow cell cycling, lower proliferation, increased expression of DNA repair, and anti-apoptosis genes.

Since most cytotoxic chemotherapeutic agents preferentially target the most proliferative cells, quiescence (or dormancy) renders CSCs insensitive to these drugs.

Recently, resistance against biological drugs through CSC involvement has also been established^[100]^. Therefore, there are many clinical failures due to the development of inherent/acquired resistance to antiangiogenic drugs, but the role of ALDH1A1 in this context is debated. Various studies have found a correlation between the expression of stemness marker and resistance to antiangiogenic drugs^[100-102]^. A critical point is that tumor angiogenesis is not only a VEGF-dependent process since there are several key factors responsible for this phenomenon, but most drugs inhibit the VEGF signaling.

### ALDH and immune evasion

Finally, the acquisition of stem-like phenotype is a putative mechanism by which CSCs develop refractoriness to current immunotherapeutic treatment strategies^[103,104]^. Resistance of CSCs to immunotherapy has been reported in a glioma model of vaccination with dendritic cells^[105]^. This is consistent with the ability of tumors to avoid the immune destruction^[2]^. Emerging literature shows that the enrichment of PD-L1 expression in CSCs contributes to CSC immune evasion^[106]^. In 2019, Castagnoli *et al*.^[107]^ showed an enrichment of PD-L1 expression in tissues from ALDH1A1 positive and triple negative breast cancer patients^[107]^. Interestingly, clinical samples and 4T1 murine mammary cells (Aldefluor+) express lower levels of antigen processing and presentation proteins (TAP-1 and TAP-2) and co-stimulatory molecules (CD80). This pattern of expression decreases the susceptibility of CSCs to immune T cell-mediated attack^[108]^.

In the complex, the interaction between CSCs and the immune system is of much interest, as their increased tumorigenicity suggests they may have enhanced immunoediting mechanisms.

## Conclusion

**Figure 1:**
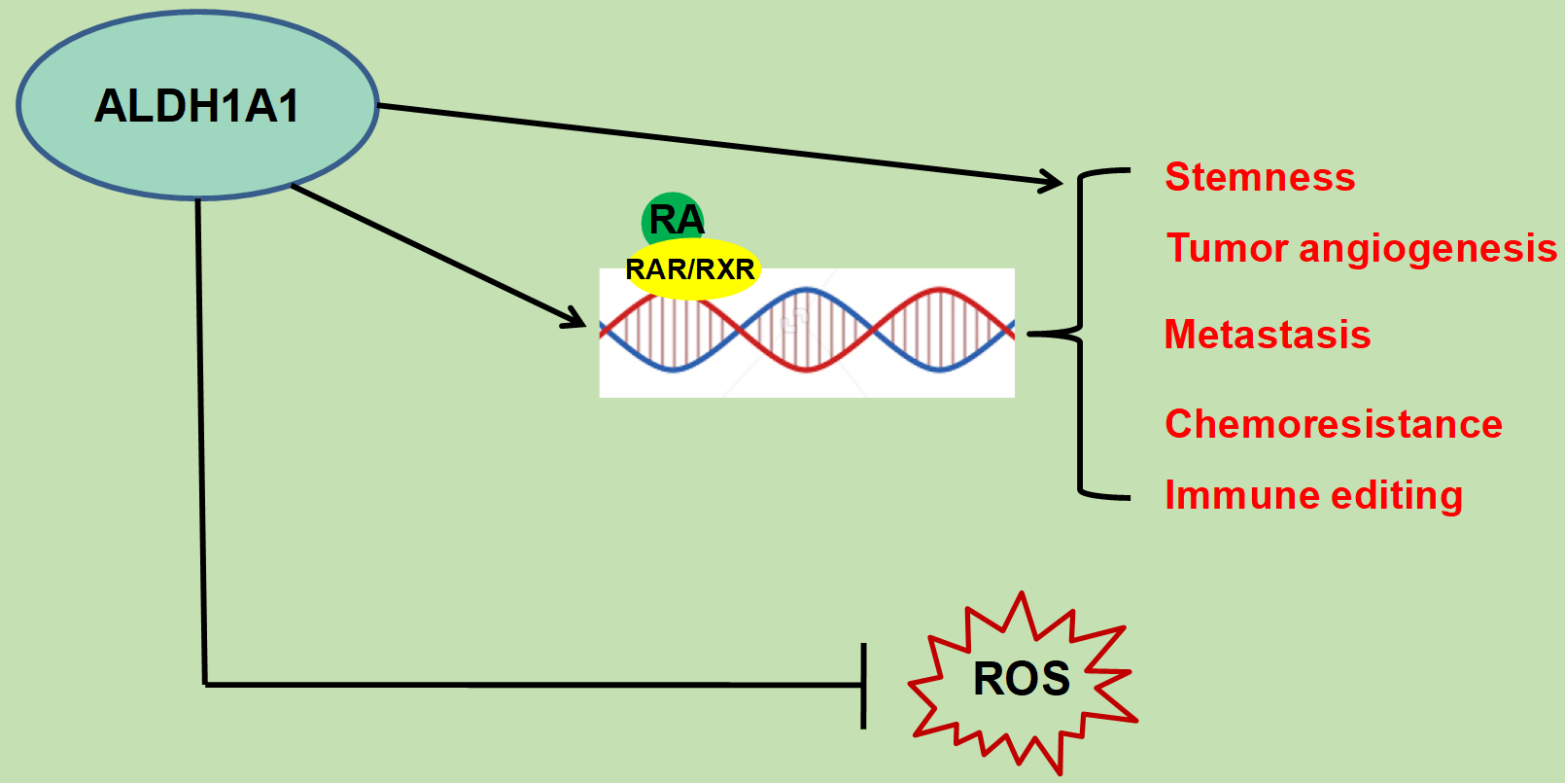
Role of ALDH1A1 in cancer cells. ALDH1A1 regulates several hallmarks of cancer, strengthening its central role in tumor progression and malignancy. ALDH1A1: Aldehyde dehydrogenase 1A1; RA: retinoic acid; RAR: retinoic acid receptor; RXR: retinoic X receptor; ROS: reactive oxygen species

The idea to develop therapeutic anti-cancer strategies that consider the heterogeneity of cancer is a medical option in the field of tumor biology and treatment. CSCs are an excellent example of tumor plasticity and the preclinical and clinical evidence suggests their importance in cancer progression, relapse, metastases, and chemoresistance^[109]^. The discovery of phenotypic switching of tumor cells into CSCs and vice versa opens new possible therapeutic options. It has been ascertained that environment and genetic alterations regulate CSC switch. ALDH1A1 is responsible for the maintenance of the stem-like state in tumor cells, in which several hallmarks of cancer are activated [Figure 1], and it is also frequently overexpressed and/or activated in more differentiated cancer cells. The switching from undifferentiated state to differentiated state is highly dynamic and the fate of stem-like state can be decided by ALDH1A1 expression/activity in tumor cells, suggesting that this enzyme might represent a concrete novel target for cancer treatment.

## Declarations

### Copyright

© The Author(s) 2020.
